# Adaptive therapy and its challenges

**DOI:** 10.1093/emph/eoag010

**Published:** 2026-05-17

**Authors:** Jinling Wu, Maximilian A R Strobl, Jacob G Scott

**Affiliations:** School of Medicine, Case Western Reserve University, Cleveland, OH, USA; Genomic Sciences and Systems Biology, Cleveland Clinic Research, Cleveland, OH, USA; Centre for Evolution and Cancer, Division of Cancer Biology, The Institute of Cancer Research, London, UK; Department of Life Sciences, Imperial College London, London, UK; School of Medicine, Case Western Reserve University, Cleveland, OH, USA; Genomic Sciences and Systems Biology, Cleveland Clinic Research, Cleveland, OH, USA; Department of Physics, Case Western Reserve University, Cleveland, OH, USA

## NAME OF PATHOLOGY/CONDITION

Cancer remains a leading cause of global mortality, with treatment failure often driven by resistance evolution. Adaptive therapy offers an evolution-informed approach to delay resistance and improve outcomes in metastatic and recurrent disease. Here, we discuss its promise, key challenges in clinical implementation, and opportunities offered by circulating tumor DNA (ctDNA) technology.

## EVOLUTIONARY PERSPECTIVES

Tumor evolution under therapy is shaped by selective pressures that drive the emergence and expansion of resistant populations. Treatment-resistant clones are either pre-existing at low frequency or emerge through *de novo*, and potentially even drug-induced, changes during treatment. Treatment may also change the ecology within the tumor to facilitate recurrence, e.g. by altering tumor vasculature. At the same time, tumor cells co-exist in a complex and heterogeneous mixture of other tumor cells and normal tissues, forming an interacting ecology in which cells compete for limited space and resources [1]. In this context, it has been proposed that in advanced cancers, aggressive treatment strategies used in current standard-of-care protocols and aimed at tumor eradication may inadvertently accelerate the expansion of resistant populations by primarily eliminating sensitive cells and thereby freeing resistant cells from competition in a phenomenon called ‘competitive release’ [2].

These insights have motivated evolutionary therapies that seek to use evolutionary and ecological principles to delay resistance and steer tumor adaptation. Among these, adaptive therapy is one of the best known and most clinically tested strategies, developed to integrate our understanding of tumors as evolving ecosystems into clinical care [2]. This approach modulates treatment intensity to preserve a subpopulation of sensitive cells and suppress resistant clones through intra-tumoral competition, thereby enabling long-term evolutionary control of resistance trajectories. This is achieved through iterative and personalized cycles, where treatment is applied to reduce tumor burden and then paused to allow sensitive cells to rebound, thereby restraining resistant populations through competition for limited resources and space [3]. Initial results in prostate cancer indicate adaptive therapy is feasible and can reduce cumulative drug use and the associated financial healthcare burden [4], while maintaining, and potentially extending, tumor control [5].

The success of adaptive therapy depends on several key evolutionary conditions. First, it is assumed that resistant clones pre-exist and cannot be fully eliminated by maximum tolerated dose strategies. Second, sensitive and resistant cells are in competition with each other for limited space and resources, and ideally, in the absence of treatment, resistant cells pay a ‘resistance cost’ so that sensitive cells have a competitive advantage during treatment breaks [6]. Third, treatment does not induce a ‘drug-tolerant’ or ‘intermediate resistant state’ in the ‘sensitive’ cells, or if it does, then these cells rapidly re-sensitize during treatment breaks [7, 8]. Adaptive therapy is now under active investigation in melanoma, prostate, ovarian, and breast cancer [5, 9–12].

However, adaptive therapy remains largely constrained to settings where one can reliably monitor tumor burden using quantitative imaging or circulating biomarkers (e.g. prostate specific antigen (PSA)). Implementation is further limited by the need for precise temporal control over drug activity, which is more feasible with targeted therapies or chemotherapy than with immunotherapies. Psychological factors also play a role, as patients may be uneasy with treatment pauses and the uncertainty of when therapy will resume. Bridging these gaps will require innovative monitoring tools and improved integration of emerging technologies, most notably ctDNA, to enhance precision and expand applicability.

## FUTURE IMPLICATIONS

A fundamental challenge in implementing adaptive therapy clinically is ensuring continuous, non-invasive monitoring of tumor evolution during treatment. To be most effective, we require not only estimates of total tumor burden, but also dynamic information about the composition and frequency of resistant versus sensitive subpopulations. Traditional approaches, including imaging modalities, offer coarse-grained readouts of tumor size or systemic activity without resolution into clonal dynamics. Although tissue biopsy can provide cellular and molecular insights, its invasive nature limits sampling frequency.

Across multiple cancers, longitudinal ctDNA profiling has emerged as a minimally invasive, dynamic biomarker that reflects both tumor burden and clonal composition. As such, it opens up promising opportunities to employ adaptive therapy even in settings without easily accessible circulating biomarkers, such as PSA. Furthermore, the safe implementation of adaptive therapy depends on continuous validation of its evolutionary premises. If competitive suppression of resistant clones is not observed, or if resistant populations expand despite treatment modulation, reconsideration of the therapeutic strategy may be necessary to avoid inadvertently facilitating resistance. In this context, ctDNA allows adaptive therapy to be operationalized as a testable evolutionary hypothesis, enabling longitudinal assessment of whether individual patients are deriving the intended benefits of competitive suppression and resistance delay.

Two clinical trials are currently exploring adaptive therapy guided by ctDNA in advanced, BRAF-mutant melanoma (NCT03543969, NCT06470880). The results from these studies will provide important insights into the feasibility of ctDNA-guided adaptive therapy and the clonal dynamics during treatment. Further settings in which ctDNA-guided adaptive therapy may be a promising option are advanced non-small cell lung cancer (NSCLC; [13]) and colorectal cancer (CRC). For example, longitudinal monitoring of ctDNA during immunotherapy for patients with advanced NSCLC has shown that ctDNA clearance correlates with radiographic response and improved progression-free and overall survival [14]. Building on these results, a follow-up study is now evaluating the use of real-time ctDNA dynamics to guide therapy escalation in those patients with a poor ctDNA response (NCT04093167). Similarly, in metastatic CRC, the CHRONOS study demonstrated that ctDNA can help to identify those patients, for whom a treatment break successfully cleared drug-resistant clones driving prior anti-EGFR progression, allowing for successful re-challenge with anti-EGFR therapy [15]. Concurrently, the IMPROVE trial demonstrated that treatment breaks during first-line treatment can extend survival and reduce toxicity, in particular in patients with left-sided disease [16]. Forthcoming ctDNA analysis from IMPROVE will further illuminate how treatment breaks shape clonal evolution and provide key insights for future adaptive therapy development in CRC.

In conclusion, adaptive therapy provides a promising framework to delay resistance by treating tumors as dynamic ecosystems guided by real-time evolutionary signals. With continued clinical innovation and cross-disciplinary collaboration, this strategy will enable oncologists to transition from rigid dosing cycles to personalized treatment decisions tailored to the tumor’s evolutionary trajectory.
